# Strengthening the AntiTumor NK Cell Function for the Treatment of Ovarian Cancer

**DOI:** 10.3390/ijms20040890

**Published:** 2019-02-19

**Authors:** Marco Greppi, Giovanna Tabellini, Ornella Patrizi, Simona Candiani, Andrea Decensi, Silvia Parolini, Simona Sivori, Silvia Pesce, Laura Paleari, Emanuela Marcenaro

**Affiliations:** 1 Department of Experimental Medicine (DIMES) and Centre of Excellence for Biomedical Research (CEBR), University of Genoa, 16132 Genoa, Italy; marcogreppi92@gmail.com; 2 Department of Molecular and Translational Medicine, University of Brescia, 25123 Brescia, Italy; giovanna.tabellini@unibs.it (G.T.); ornella.patrizi@unibs.it (O.P.); silvia.parolini@unibs.it (S.P.); 3 Department of Earth Science, Environment and Life (DISTAV), University of Genoa, 16132 Genoa, Italy; candiani@unige.it; 4 Division of Medical Oncology, Galliera Hospital, 16126 Genoa, Italy; andrea.decensi@galliera.it; 5 Wolfson Institute of Preventive Medicine, Queen Mary University of London, London E1 4NS, UK; 6 Department of Experimental Medicine (DIMES), University of Genoa, 16132 Genoa, Italy; silvia.pesce@unige.it; 7 A.Li.Sa., Liguria Health Authority, 16121 Genoa, Italy; laura.paleari@regione.liguria.it

**Keywords:** ovarian cancer, NK cells, immunotherapy, immune checkpoint, PD-1, activating receptors, B7-H6, antitumor activity, hormone therapy, adoptive therapy

## Abstract

The crosstalk between cancer cells and host cells is a crucial prerequisite for tumor growth and progression. The cells from both the innate and adaptive immune systems enter into a perverse relationship with tumor cells to create a tumor-promoting and immunosuppressive tumor microenvironment (TME). Epithelial ovarian cancer (EOC), the most lethal of all gynecological malignancies, is characterized by a unique TME that paves the way to the formation of metastasis and mediates therapy resistance through the deregulation of immune surveillance. A characteristic feature of the ovarian cancer TME is the ascites/peritoneal fluid, a malignancy-associated effusion occurring at more advanced stages, which enables the peritoneal dissemination of tumor cells and the formation of metastasis. The standard therapy for EOC involves a combination of debulking surgery and platinum-based chemotherapy. However, most patients experience disease recurrence. New therapeutic strategies are needed to improve the prognosis of patients with advanced EOC. Harnessing the body’s natural immune defenses against cancer in the form of immunotherapy is emerging as an innovative treatment strategy. NK cells have attracted attention as a promising cancer immunotherapeutic target due to their ability to kill malignant cells and avoid healthy cells. Here, we will discuss the recent advances in the clinical application of NK cell immunotherapy in EOC.

## 1. Overview on Epithelial Ovarian Cancer

Epithelial ovarian cancer (EOC) is an endocrine-related neoplasm and it is classified as a rare cancer both in the portal for rare diseases and orphan drugs Orphanet (http://www.orpha.net/ORPHA398934) and in the National Institutes of Health (NIH) Register, Genetic and Rare Diseases (GARD) (https://rarediseases.info.nih.gov/). Most EOCs are diagnosed at an advanced stage, which accounts for the high mortality rate associated with this disease. The new World Health Organization (WHO) Classification of Ovarian Cancer takes the recent findings on the origin, pathogenesis and prognosis of different ovarian cancer subtypes into account. The tubal origin of hereditary and some non-hereditary high-grade serous cancers is mentioned in contrast to the previous theory of mesothelial origin of tumors while seromucinous tumors represent a new entity [1]. Several studies over the past decade have demonstrated that histological grade is one of the most important prognostic factors in EOC, having found important differences in the molecular and clinical characteristics of a low-grade serous carcinoma of the ovary (LGSCO) compared with a high-grade serous carcinoma of the ovary [2,3,4,5,6,7,8,9,10,11,12,13]. LGSCO represents approximately 10% of all serous ovarian carcinomas that are diagnosed at a younger age and individuals diagnosed with LGSCO experience a longer overall survival (OS) than those with high-grade disease [14]. Despite these differences, most women with EOC have been treated identically in the last three decades independently of the histological grade of their tumors. A growing body of research has questioned this strategy. Recent advances in the understanding of the tumor heterogeneity, including refinement in pathologic criteria, elucidation of molecular and genetic tumoral differences as well as disparate responses to treatment with chemotherapy, have led to the initiation of separate clinical trials according to epithelial histology subtypes through the NRG Oncology (Gynecologic Oncology Group) Rare Tumor Committee [14,15,16]. Surgery is effective in most cases of early stage EOC (Federation Internationale des Gynaecologistes et Obstetristes—FIGO stage I-IIA) with a 5-year survival rate of around 90% [17]. After surgery, the treatment of choice for advanced EOC (stage IIB-IV) is platinum-based chemotherapy (CT) [18]. Conversely, women with LGSCO exhibit poor response rates to conventional chemotherapy and remain at high risk of recurrence and cancer-related death, especially in the setting of advanced stage disease [2,14,15,16,19,20,21].

## 2. Epithelial Ovarian Cancer: A Focus on Tumor Escape Mechanisms Impairing NK Cell Function

EOC is the 7th cause of death among women with malignancies worldwide and the leading cause of death from gynecological cancers (https://www.uicc.org/new-global-cancer-data-globocan-2018) [22]. EOC spreads predominantly in the peritoneal cavity and is often accompanied by a massive production of (malignant) ascites. This increasing volume of ascites can generate a favorable tumor microenvironment, enabling the characteristic patterns of transcoelomic tumor spread in ovarian cancer [23]. This ascites is rich in tumor-promoting soluble factors [24,25], extracellular vesicles [26] and detached cancer cells [27] as well as large numbers of immune cells, such as Natural Killer (NK) cells, T cells and tumor-associated macrophages (TAMs). After being influenced by the tumor microenvironment, these cells are unable to defend our body against tumors but instead cooperate with resident host cells to support tumor progression and immune evasion [25,28].

The malignant transformation of normal cells comes from a multifactorial process, resulting in genomic instability [29] and a modification of immunosurveillance mechanisms that induces tolerance. To reach this state, tumors develop different strategies during their evolution to escape the immune response: (i) the secretion of immunosuppressive cytokines or soluble tumor-derived inhibitory factors [24,25]; (ii) the induction of coinhibitory receptors (e.g., immune checkpoints) [30,31]; or the dampening of costimulatory receptors [25,32,33] on infiltrated lymphocytes.

NK lymphocytes represent one of the most efficient cellular mechanisms by which the immune system can recognize and kill tumors. With an array of receptors evolved to sense cellular alterations, NK cells provide early protection against cancer cells by producing cytokines and chemokines in addition to collaborating with other immune cells and exerting direct cytolytic activity [34,35,36]. In particular, NK cells express a repertoire of activating and inhibitory receptors. The integration of signaling generated by these receptors will determine the activation status of these cells [37]. No single receptor dominates as synergistic signals from combinations of receptors are instead integrated to activate or inhibit natural cytotoxicity and cytokine production. A prevalence of inhibitory signals induces the blocking of NK cell functions while a prevalence of activating signals leads to the activation of NK cells and consequently the killing of NK-susceptible target cells. Despite the fact that NK cells display potent cytolytic activity against tumor cells in vitro, this functional capability may be strongly impaired by the TME. Indeed, ascites not only contain large numbers of growth factors and cytokines that are able to promote the proliferation of tumor cells but they can also suppress the function of otherwise normal immune effectors, including NK cells [25,30,32,33].

Fresh NK cells (CD56^+^CD3^−^) isolated from the ascites fluid are found in relatively high concentrations compared to peripheral blood and in particular, they result enriched in CD56^bright^ NK cells. However, although they are present in a large number, they display functional impairment [38,39]. In this regard, it has been shown that the presence of IL-18 and TGFβ in ascites can induce a strong downregulation of CD16 on NK cells, resulting in diminished antibody-dependent cell-mediated cytotoxicity (ADCC) against autologous tumor cells [25,32,40,41]. In addition, TGFβ can also contribute to the downmodulation of the NKp30 and NKG2D NK cell activating receptors [42] thus impairing NK cell-mediated natural cytotoxicity. NKG2D expression may be also impaired by macrophage migration inhibitory factor (MIF), which is another soluble factor that is detectable in ascites [43]. Recently, additional mechanisms of tumor escape have been described, including the ability of tumor cells to release soluble forms of activating NK cell receptor ligands. The chronic receptor–ligand interaction may dampen the surface expression of the activating NK receptors, thus affecting the ability of NK cells to kill tumor cells that express ligands for those receptors. In particular, ovarian cancer cells may release a soluble form of B7-H6, the main NKp30 ligand, leading to the loss of NKp30 expression on NK cells in the TME. A high amount of soluble B7-H6 is correlated with a greater downmodulation of NKp30. These NK cells display impaired IFN-γ production and cytolytic function, thereby showing poor NK cell-mediated elimination of B7-H6^+^ ovarian cancer cells [25]. Consistent with these observations, a lower level of B7-H6 expression is correlated with a better OS and reduced metastasis and cancer progression in ovarian cancer patients [44]. Similar results have also been shown for NKG2D. Indeed, soluble MIC-A and MIC-B, two NKG2D ligands that are released by tumor cells in the TME, can downmodulate the expression of this activating receptor, which is an event associated with an adverse clinical outcome [33]. Finally, the activating NK cell receptor DNAM-1 can also be downmodulated by the chronic exposure to the ligand expressed on the surface of ovarian tumor cells [32].

Recently, the attention of researchers has been focused on other molecules expressed on immune cells, which are the inhibitory receptors that are defined as immune checkpoints. Under healthy conditions, these receptors maintain self-tolerance and modulate the duration and amplitude of immune responses upon the recognition of specific ligands on normal cells in order to prevent collateral tissue damage. However, it is now known that tumor cells may coopt these inhibitory receptors in order to avoid immune surveillance [45]. For this reason, immune checkpoint expression and engagement are now known as one of the major mechanisms related to tumor resistance or escape from the immune system-mediated attack. In fact, during tumorigenesis, cancer cells often express ligands to bind these receptors and induce immune suppression. NK cells are equipped with several inhibitory receptors. In particular, NK cell immune checkpoints include the well-known HLA-class I-specific receptors KIR, LIR-1 and NKG2A, but also non-HLA class I-specific receptors, such as PD-1. This receptor was originally discovered on cytolytic T cells and was found to exert a sharp inhibitory effect on their proliferation, cytokine production and antitumor activity upon binding to its ligands (PD-L1 and PD-L2). Thus, by expressing PD-1 ligands, tumors have evolved a remarkable mechanism to avoid T cell-mediated surveillance of cancer. A recent discovery from our research group showed that PD-1 is also expressed on a discrete fraction of NK cells in patients with EOC [30]. In vitro experiments showed that these cells display a strongly reduced capacity to kill PD-L^+^ tumor cells as well as impaired release of interferon-γ (IFN-γ) and tumor necrosis factor-α (TNF-α) cytokines after stimulation with the same PD-L^+^ tumor targets. Remarkably, these impaired effector functions induced by PD-1/PD-L interaction could be partially reverted by mAbs specific for PD-L1/PD-L2 [30].

Despite the fact that the functional capability of NK cells may be strongly impaired by the TME, NK-based immunotherapeutic approaches for treatment of tumors have garnered attention, primarily for NK cell capability of killing malignant cells without toxicity towards healthy cells.

## 3. State-of-the-Art Therapies Targeting Ovarian Cancer

Despite the prognosis of early-stage ovarian cancers being favorable with approximately 90% of patients surviving 5 years after diagnosis [46], more than 70% of patients are diagnosed with advanced disease (FIGO stage III-IV) due to the lack of sensitive screening during the early stages [47]. Many of these patients initially benefit from integrated surgery and platinum/taxane-based chemotherapy although recurrence develops in nearly 90% of cases. Furthermore, 70% of patients with advanced disease succumb to tumor relapse within less than five years [48,49]. This poor prognosis is attributed to the development of drug-resistance through the selection of chemoresistant clones, intraperitoneal spreading of tumor cells and formation of metastatic lesions and tumor relapse. In this regard, there is a clear urgent need for alternative treatments to improve the clinical outcome of patients with advanced ovarian carcinoma.

The currently available effective approaches for recurrent ovarian cancer include cytokine therapy, adoptive transfer of NK cells, hormone therapy and antibody-based immunotherapy [50].

### 3.1. Cytokine Therapy

Early clinical trials in ovarian cancer patients aimed to improve the antitumor activity of immune cells through intraperitoneal injections of different biologic products, including *Bacillus Calmette-Guerin*, *Corynebacterium parvum* and an attenuated strain of influenza virus [51,52]. These treatments had limited clinical responses mainly due to the small number and heterogeneity of study participants.

Another immunotherapeutic approach for ovarian cancer is the intraperitoneal administration of cytokines to potentiate an autologous antitumor response in vivo. In this context, the results of several clinical trials evaluating intraperitoneal therapy with IL-2 alone or in combination with other therapies demonstrated that cytokine therapy was generally well tolerated and may improve lymphocyte and NK cell counts. However, cytokine therapy had variable levels of success and was mainly dependent on the remaining tumor burden before the start of therapy [53,54,55,56,57]. IL-15, which is similar to IL-2, can strongly increase NK cell numbers and may also enhance NK cell function in the ovarian cancer setting [58,59]. Currently, several clinical trials evaluating IL-15 are ongoing [60]. In this regard, it has been demonstrated that monomeric IL-15 or the IL-15 superagonist fusion complex, ALT-803, potently increases the function of ascites-derived NK cells [61,62].

### 3.2. Adoptive Therapy of Immune Cells

An additional approach in ovarian cancer involves the adoptive transfer of immune cells isolated from the peripheral blood of patients, which was activated with various cytokines and subsequently infused back into the same patient. This aims to improve the autologous antitumor responses [63,64].

The early adoptive transfer of autologous lymphokine-activated killer (LAK) cells with a high dose of IL-2 demonstrated limited clinical responses with high rates of peritoneal fibrosis [65,66,67]. Cytokine-induced killer (CIK) cells (derived again from peripheral blood and stimulated with antiCD3 mAbs, IFN-γ and IL-2) [68] demonstrated enhanced cytotoxic activity compared to LAK cells against ovarian cancer [69]. Recently, promising results were obtained by a phase III clinical trial in which the adoptive transfer of autologous CIK cells after primary debulking surgery and adjuvant carboplatin/paclitaxel chemotherapy was assessed [70]. These studies suggest that allogeneic NK cell therapy is feasible although further efforts that will generate novel strategies to increase in vivo NK cell persistence and expansion after adoptive transfer are needed.

In this regard, it has been reported that adaptive NK cells induced by different cytokines (IL-12, IL-15, IL-18) display both in vitro and in vivo enhanced functionality and persistence against ovarian cancer. Notably, this higher NK activity was detectable even upon exposure to ascitic fluid, thus suggesting its capability to circumvent the immunosuppressive nature of ovarian cancer TME [71].

In addition, the ex vivo inhibition of GSK3 kinase in peripheral blood induces an enrichment of mature adaptive NK cells from cytomegalovirus positive donors and enhances their cytokine production and ADCC when exposed to tumor cells [72]. A phase I clinical trial using the product generated from this method has been started at the University of Minnesota (NCT03213964).

Many NK cell-adoptive therapies against malignancies are currently in clinical practice, including hematopoietic stem cell transplantation. NK cell infusions can provide safe and effective immunotherapy against tumor relapse [73]. Usually, these therapies use “adult” cell populations, such as hematopoietic stem cells (HSCs) from bone marrow (BM), peripheral blood (PB) or cord blood (CB) cells. Recent studies demonstrated the ability of “non-adult” human pluripotent stem cells (h-PSCs) to generate NK cells. The proportion of mature and functional cytolytic NK cells is higher from the hPSCs-derived progenitor cells [74,75]. This probably allows hPSC-NK cells to mediate an increased antitumor response both in vitro and in vivo, thus providing an alternative source of cells for the immunotherapy of different type of tumors, including ovarian cancer.

### 3.3. Hormone Therapy in Ovarian Cancer

A putative direct action of gonadal steroids on ovarian carcinogenesis has been suggested, which was supported by findings of mRNA transcripts and translated proteins of Estrogen receptor (ER) and Progesterone receptor (PgR) in both normal ovarian tissue and malignant ovarian tumors. A direct action of estrogen on EOC growth, metastasis and progression has been demonstrated through different pathways, including: (i) tumor production of vascular endothelial growth factor (VEGF) via ER signaling (direct pathway); and (ii) increased tumor–endothelial cell migration via mitogen-activated protein kinase (MAPK) signaling (indirect pathway) [76]. PgR activation induces apoptosis, cell cycle arrest and senescence in ovarian cancer cells, which strongly suggests the modulation of PgR levels and/or activity as a form of endocrine treatment of EOC [77]. In a recent large observational study, Sieh et al. (2013) found a high positivity for ER/PgR (60–80%) in high grade EOC, with a prognostic value that is associated with significantly improved survival [78]. Moreover, it has been shown that ER and PgR are twice as likely to be expressed in LGSCOs than in high-grade serous carcinomas of the ovary [15]. Since hormone therapy may become a viable and extremely cost-effective option for the treatment of EOC, we meta-analyzed 53 clinical trials to assess the Clinical Benefit Rates (CBRs) and deaths in EOC after hormone therapy [79]. Overall, we found a summary estimate of CBR (SCBR) of 41% (95% CI = 0.34–0.48) and 46% (95% CI = 0.34–0.57) for ER^+^ and/or PgR^+^ tumors while this CBR was 40% (95% CI = 0.29–0.51) in platinum resistant tumors. In particular, subgroup analyses by type of hormonal treatment showed a SCBR for aromatase inhibitors of 39% (95% CI = 0.29–0.50) and the highest clinical benefit of 43% (95% CI = 0.30–0.56) with tamoxifen, which is a selective ER modulator (SERM) that produces antiestrogen effects through competitive inhibition of the receptor itself. Explorative analyses according to line of treatment and histological grade were hampered by the low numbers although there was a tendency to find a greater effect in first-line (adjuvant setting) and LGSCO. Specifically, hormone therapy was associated with a significant reduction of mortality in LGSCO (HR = 0.66, 95% CI = 0.47–0.93) [79]. In a recent retrospective study by the MD Anderson group, 203 women with stage II–IV LGSCO who received hormonal maintenance therapy following primary treatment had a better outcome compared with those on only chemotherapy [80]. The median PFS was 26.4 months with surveillance and 64.9 months with hormonal therapy (*p* < 0.001). Regarding OS, the subgroup analysis by disease at the end of adjuvant chemotherapy (disease free vs. persistent disease) showed a positive result in favor of hormonal therapy in both subgroups (in women who were disease-free, OS was 191.3 months vs. 106.8 months; in women with persistent disease, median OS was 83.3 months vs. 44.4 months). A stratified log-rank test adjusted for disease status determined that *p* = 0.014. A second study, performed at Johns Hopkins Hospital in conjunction with the Cleveland Clinic during the same period [81], utilized hormonal monotherapy after primary cytoreductive surgery and letrozole, which is an aromatase inhibitor that prevents estrogen synthesis, which was the predominant hormonal therapy used in 55.6%. The three-year PFS was 79.0% and three-year OS was 93.1%.

Interestingly, it has been shown that murine NK cells expressed ERα and ERβ and that 17β -estradiol elicited a significant decrease in NK activity in both wild type and ERα-deficient mice (*p* < 0.001). This suggests that ERβ is involved in mediating the actions of estrogen on NK cell activity and increasing the potential for therapeutic modulation of NK cell activity [82]. Moreover, it has been demonstrated that the antitumor activity of NK cells in human is modulated by estrogen. The most effective treatment for breast cancer patients, the antiestrogen tamoxifen, has been shown to stimulate host NK cell activity and metastasis in xenograft models [83,84,85]. These data are taken into account with a view to design new therapeutic treatments for patients based on the use of NK cells in EOC therapy. In this regard, an innovative therapeutic approach against EOC could be based on the improvement in NK cell antitumor function by combining immunotherapy and hormone therapy. 

### 3.4. Immunotherapy

The approaches using antibody (Ab)-based immunotherapy have also been explored and some are heading towards being used in the clinic. The mAbs generated to induce/amplify an antitumor response can function through different mechanisms, including the opsonization/activation of ADCC and blockage of immune checkpoints.

The first treatment that aims to target the previously identified ovarian cancer-associated antigens [including NY-ESO-1, CA 125 (MUC16), MUC1 and epithelial cell adhesion molecule (EpCAM)] with mAbs [55] or with new engineered bispecific antibodies and bispecific/trispecific killer engagers (BiKEs or TriKEs), which are molecules that crosslink tumor cells antigens (e.g., EpCAM) with CD16 on NK cells, thus activating/enhancing ADCC [86,87]. A fully humanized TriKE, which utilizes a modified IL-15 to crosslink the antiCD16 scFv and EpCAM scFv, not only sustains ADCC activity, but also mediates NK expansion, cytokine production and survival via IL-15 [88]. A TetraKE construct was recently engineered to simultaneously target EpCAM and CD133 bearing cells [89].

Given the results of GOG-0218 [90] and ICON7 [91] trials, bevacizumab, a mAb that functions in a non-immune-mediated manner by blocking VEGF, has recently been approved in combination with CT as a first-line treatment of advanced EOC (stage IIIb–IV) although an OS benefit has not been demonstrated [90,91]. Despite the use of bevacizumab, the disease prognosis remains poor as the European mean age-standardized 5-year OS was only 37.6% for women diagnosed between 2000 and 2007 [92] and the median OS in ICON7 in the CT plus bevacizumab arm was 58 months [91]. Despite this recent progress, not all patients may benefit from bevacizumab and the cost/benefit ratio of this drug remains unclear [93].

Another promising approach is the infusion of antitumor lymphocytes that were previously engineered with chimeric antigen receptors (CARs). These studies are mainly focused on cytotoxic T cells but recently, these technologies have also been applied to NK cells. In a recent study, the effect of the chemotherapeutic agent cisplatin in association with a CAR-based immunotherapeutic approach was evaluated to improve the clinical efficacy of ovarian cancer therapies. In particular, a lentiviral vector encoding a third-generation anti-CD133-CAR was generated and transduced in NK92 cells. This combined clinical approach led to a strong killing effect against ovarian cancer stem cells [94]. Efforts to generate additional ovarian cancer specific NK chimeric antigen receptors are ongoing [95].

In high-grade serous ovarian carcinomas, the most common histological type of ovarian cancer, almost 15% of women harbor germline BRCA mutations [96]. A recent study has shown that BRCA-mutated high-grade serous ovarian carcinomas have a high mutational load and there are more tumor-specific neoantigens [97,98] that recruit and activate tumor-infiltrating T lymphocytes. This induces a compensative upregulation of the immune checkpoint PD-1 on the surface of T cells. Immune checkpoints are inhibitory pathways that serve to prevent self-tissue damage under healthy conditions. During tumorigenesis, cancer cells often express ligands for this (PD-L1 and PD-L2) and other immune checkpoints, which downregulates T cell activity and induces immune suppression. In the case of BRCA-mutated ovarian carcinoma, the inhibition of the PD-1/PD-L1 pathway may induce cytotoxic lymphocyte activity against cancer cells. A recent clinical trial has shown antitumor activity against tumors with mismatch repair deficiencies after treatment with immune checkpoint inhibitors (including nivolumab) [99,100]. In addition, a recent study suggests that nivolumab monotherapy in women with BRCA gene mutations, high-grade serous histology and recurrent Mullerian cancer may be an effective and well-tolerated salvage therapy [101]. Thus, targeting the PD-1 pathway with a checkpoint inhibitor is an attractive approach in hypermutated tumors. This indicates that the immune system is protective against ovarian cancer and thus adjuvant immunotherapy post-surgery and with chemotherapeutics could be effective for preventing relapse and extending survival [102,103].

Current efforts for a NK cell based therapy are mainly based on strategies that manipulate the function of inhibitory receptors. In this context, it has been recently demonstrated that ascites-associated NK cells can also express high levels of PD-1, with this expression potentially impairing the antitumor activity of these innate effectors [30]. These findings may extend the therapeutic use of anti-PD-1 mAbs to unleash the cytotoxic potential of NK cells against NK susceptible malignancies, including ovarian cancer cells. Moreover, the combined antibody-mediated blocking of multiple inhibitory checkpoints on NK cells, including NKG2A, KIR and PD-1, by triggering their ability to kill tumor cells is likely to facilitate the uptake of novel/additional tumor antigens by antigen presenting cells and subsequent massive recruitment of antigen-specific T lymphocytes [104]. In this context, a phase-1 dose-ranging study of anti-NKG2A (IPH2201) in patients with gynecologic malignancy, including high-grade serous ovarian/fallopian tube or peritoneal carcinoma, cervical cancer (squamous cell carcinoma) or endometrial cancer (adenocarcinoma), that is advanced/metastatic/recurrent or unresectable and for which no curative therapy exists is ongoing (NCT02459301, Canadian Cancer Trials Group, Collaborator: Innate Pharma).

Future research is needed to clarify the effects that checkpoint inhibitors have on the NK cell response and the potential to enhance adoptive NK cell immunotherapy in ovarian cancer [71].

A combination of these different techniques of NK cell-based immunotherapy hold great potential and may represent an effective weapon against ovarian cancer after primary cytoreductive surgery and adjuvant chemotherapy.

## 4. Conclusions

Based on the evidence of the high frequency of persistent tumors at the completion of primary postoperative chemotherapy and the high relapse rate for women with advanced EOCs treated with standard surgery/chemotherapy, there is a clear medical need for identifying a management strategy that results in improved outcomes. Further studies are needed to provide new insights on the immunological mechanisms involved in the development of certain forms of ovarian cancer, which will be crucial for the development of new immunotherapeutic strategies (e.g., simultaneous blockage of different immune checkpoints, including hormone receptors, or infusion of antitumor lymphocytes previously engineered with CARs).

The recent evidence for a strong prognostic effect of ER in a large proportion of newly diagnosed EOC offers a therapeutic target for a very cost-effective precise medical approach.

Increasing our understanding of the mechanisms of NK cell activation, including those regulated by different molecular checkpoints and hormone receptors, and the identification of new tumor biomarkers will provide a firm basis for how to optimize NK cell reactivity against cancer. This knowledge can be applied to the development of an optimal design for cancer immunotherapy by targeting NK cells either alone or in combination with other therapies.
